# Design and Screening of the Peptide SAMP-12aa Derived from LL-37, Which Exhibits Anti-*H. Pylori* Activity and Immunomodulatory Effects

**DOI:** 10.3390/molecules31061002

**Published:** 2026-03-17

**Authors:** Jianliang Lu, Qingyu Wang, Meisong Qin, Jinfeng Dou, Youyi Xiong, Xiaolin Zhang

**Affiliations:** The Department of Pharmacy, School of Biomedical and Health Sciences, Anhui Science and Technology University, Fengyang 233100, China; lujianliang03@126.com (J.L.); qingyuwang99@126.com (Q.W.); qinms@ahstu.edu.cn (M.Q.); doujf@ahstu.edu.cn (J.D.); xiongyy@ahstu.edu.cn (Y.X.)

**Keywords:** antimicrobial peptide, cell selectivity, anti-*H. pylori* activity, regulatory T cells, transcription factor Foxp3, molecular docking, immunomodulatory effects

## Abstract

The appearance of antibiotic-resistant strains of *Helicobacter pylori* (*H. pylori*) is leading to a decreased eradication rate of *H. pylori* infection. There is an urgent need to find new agents with antimicrobial mechanisms different from those of antibiotics, with therapeutic potential to clear colonization of *H. pylori* in the stomach. Some antimicrobial peptides (AMPs) possess bactericidal activity by enhancing the permeability of the outer membrane and damaging the integrity of the cell membrane. Bacteria are not susceptible to drug resistance through this antimicrobial mechanism. In this study, 28 short peptides containing 12 amino acid residues were designed based on nine amino acid fragments (KRIVQRIKD) from human cathelicidin LL-37, which is stable in gastric juice, and 3 amino acids were added at the C-terminus of the peptide. These designed peptides were not digested and degraded by pepsin at low pH values. The peptides were predicted using the online tool platform. Then, the strongest antimicrobial peptide, named SAMP-12aa (KRIVQRIKDVIR), was screened from 28 short peptides. Further studies found that SAMP-12aa retained anti-*H. pylori* activity after incubation in simulated gastric juice. The MIC and MBC of SAMP-12aa were 8 μg/mL and 32 μg/mL, respectively. SAMP-12aa showed good bactericidal kinetics. SAMP-12aa was found to have cell selectivity, penetrating and damaging bacterial cell membranes and exhibiting almost no toxicity to human cells at a relatively high concentration (128 μg/mL). Regulatory T (Treg) cells express CD25^High^ with immunosuppressive activity that induces immune tolerance in response to *H. pylori*. Molecular docking prediction revealed that SAMP-12aa could target the active center of Foxp3. Flow cytometry analysis revealed that SAMP-12aa can inhibit Foxp3 activity and downregulate CD25 protein expression on CD4^+^ T cells, thereby reducing the development and differentiation of CD4^+^Foxp3^+^CD25^High^ Treg cells with immunosuppressive effects. Further research revealed that the levels of the cytokine interferon-γ (IFN-γ), which activates CD8^+^ T-cell activity, were significantly elevated, and the levels of transforming growth factor-β (TGF-β), which inhibits CD8^+^ T-cell activity, were significantly reduced. The results of this study reveal that SAMP-12aa not only possesses antibacterial activity but also has immunomodulatory effects.

## 1. Introduction

*H. pylori* infection can cause clinical symptoms of chronic gastritis, peptic and duodenal ulcers, gastric cancer, and mucosa-associated lymphohistoma. The World Health Organization has classified *H. pylori* as a class I carcinogen. People with a high infection rate of *H. pylori* often have a greater risk of gastric cancer than those without infection [1,2]. Gastric cancer has become the second leading cause of mortality after lung cancer in China. The abovementioned situation of *H. pylori* infection is related to the long-term colonization of *H. pylori* in the stomach. A proton pump combined with two triple-antibiotic and quadruple-bismuth therapies is the standard treatment for *H. pylori* infection. However, *H. pylori* is becoming resistant to a variety of antibiotics, and the cure rate of *H. pylori* is decreasing [3]. The development of new antimicrobial compounds that can kill antibiotic-resistant *H. pylori* strains is needed. Among known compounds, antimicrobial peptides (AMPs) can be suitable candidates [4,5]. LL-37 (a cationic antimicrobial peptide) is a human antimicrobial protein derived from neutrophils and various epithelial cells, and its molecular weight is 18 kDa. LL37 is a member of the human cathelicidin family, and its amino acid residues are cut from the carboxyl end of hCAP18 [6]. LL-37 has a strong antibacterial effect on Gram-positive and Gram-negative bacteria. The study found that LL-37 not only has a stronger bactericidal effect on wild *H. pylori* strains, but also has a stronger bactericidal effect on antibiotic-resistant *H. pylori* strains [7,8]. Unfortunately, the acidic environment of the stomach and pepsin have a great influence on its anti-*H. pylori* activity. Mass spectrometric analysis showed that the integrity of LL-37 was completely lost after it was warmed in simulated gastric juice. As a result, its original anti-*H. pylori* activity was almost lost [8]. Interestingly, a short peptide, KRIVQRIKDFLR-NH2 (named KR-12), from the 18th to 29th amino acid residues of LL-37 exhibited stronger antimicrobial activity [9]. Unfortunately, this short peptide was not stable in the presence of gastric acid and pepsin. A shorter peptide with nine amino acids, KRIVQRIKD-NH2 (named KR-9), was determined not to be degraded by pepsin under Ph = 1.3 and pH > 2 conditions based on an online network: https://web.expasy.org/peptide_cutter/ (accessed on 20 September 2024). The study showed that KR-12 is the smallest LL-37-derived short peptide that possesses antimicrobial activity identified using a structure–activity relationship study [10]. KR-9 has no antimicrobial activity, including that against *H. pylori*, based on our antibacterial tests and previous research [10]. Since the peptide with 12 amino acid residues could maintain stronger antimicrobial activity, we added three amino acid residues at the C-terminus of KR-9 to form each peptide with 12 amino acid residues and designed 28 peptides in this study. To ensure that the 28 designed peptides were stable at low pH values and in the presence of pepsin, an online network tool https://web.expasy.org/peptide_cutter/ (accessed on 20 September 2024).was used to conduct the analysis. Then, the strongest antimicrobial peptide, KRIVQRIKDVIR, named SAMP-12aa, was identified from the screening of the 28 peptides. SAMP-12aa was further confirmed to retain bactericidal activity and bactericidal kinetic activity in simulated gastric juice. The bactericidal mechanism of SAMP-12aa was explored by detecting the effects of SAMP-12aa on the permeability of the outer membrane and the integrity of the inner membrane. We further evaluated whether the resistant strains could be induced by SAMP-12aa and its activity against antibiotic-resistant *H. pylori*. The safety of SAMP-12aa in clinical application was studied by examining its hemolytic activity on human erythrocytes and human gastric adenocarcinoma cells. Our results indicated that a short peptide of SAMP-12aa designed based on LL-37 retained bactericidal activity and bactericidal kinetics activity in simulated gastric juice. Drug-resistant strains could not be induced by SAMP-12aa, and it possessed activity against antibiotic-resistant *H. pylori*. We found that its bactericidal mechanisms were based on the enhancement of the permeability of the outer membrane and damage to the integrity of the inner membrane. SAMP-12aa exhibited good selective toxicity to prokaryotic cells and almost no toxicity to eukaryotic cells.

Statistics have revealed that the presence of *H. pylori* in the stomach is a high-risk factor for gastric cancer [11]. A quadruple therapy of two antibiotics plus proton pump inhibitors and bismuth agents is an effective method for treating *H. pylori* infection [12]. Unfortunately, *H. pylori* has developed resistance to antibiotics, leading to treatment failure. We explore a new therapeutic approach for *H. pylori* infection using antimicrobial peptides in this study. However, the best prevention and treatment measures for *H. pylori* infection involve immune system-mediated clearance of *H. pylori* colonization in the stomach. Indeed, among individuals infected with *H. pylori*, bacterial colonization in the stomach of some individuals can be eliminated by their own immune systems, whereas other infected individuals exhibit clinical symptoms or become asymptomatic carriers who harbor the bacteria for life [13]. The underlying mechanism has been extensively studied and reported [14,15,16,17,18,19], and studies have shown that *H. pylori* infection induces severe inflammatory responses in the early stages, which can activate the immune system to generate humoral and cellular immune responses that are beneficial for clearing the colonization of *H. pylori* in the stomach. Further research has shown that the virulence factors of *H. pylori* induce the expression of forkhead box protein P3 (Foxp3) in CD4^+^Foxp3^-^ T cells in gastric tissue [15,16,17]. Foxp3 is a transcription factor that regulates CD25 protein expression, which activates CD4^+^ T cells to express the CD25 protein at high levels and become Treg cells of CD4^+^Foxp3^+^CD25^+^, especially CD4^+^Foxp3^+^ CD25^High^ Treg cells, which have immunosuppressive effects [20]. Treg cells have a certain protective effect on gastric tissue damage caused by severe immune inflammatory responses induced by *H. pylori* [21,22]. However, this does increase the load of *H. pylori* in the stomach [20]. This results in the failure of the immune-mediated clearance of *H. pylori*. *H. pylori* infection can induce the production of antibodies by humoral immunity. All individuals infected with *H. pylori* had high titers of antibodies in their serum during physical examinations; however, the antibodies were unable to clear the colonization of *H. pylori* in the gastric mucosa. The reason is that *H. pylori* survive within the epithelial cells of the gastric mucosa, and antibodies cannot fully interact with pathogenic bacteria to produce effective immune responses. Therefore, the cellular immunity of activated CD8^+^ T cells is an effective pathway for clearing *H. pylori* colonization in the stomach [14]. Research has shown that CD4^+^Foxp3^+^CD25^High^ Treg cells induce cytotoxic T lymphocyte-associated antigen 4 (CTLA-4) protein expression [20]. The second signal for the activation of CD8^+^ T cells is the binding of CD28 protein on the surface of CD8^+^ T cells to CD80/CD86 protein on antigen-presenting cells [23]. However, compared with CD28, CTLA-4 protein can also bind to CD80/CD86 protein with a higher affinity. CTLA-4 protein becomes an inhibitor of CD8^+^ T-cell activation [24]. Therefore, CD4^+^Foxp3^+^CD25^High^ Treg cells can inhibit the activation of effector CD8^+^ T cells, leading to the long-term colonization of *H. pylori* in the stomach. Hori s et al. [25] and Fontenot JD et al. [26] reported that the transcription factor Foxp3 plays a key role in the upregulation of Treg cell development. Therefore, an effective way to relieve the immune tolerance induced by *H. pylori* may involve inhibiting Foxp3 transcription activity and downregulating the expression of the CD25 protein, thereby decreasing the expression level of the CTLA-4 protein, which is beneficial for CD8^+^ T-cell activation and the clearance of *H. pylori* colonization in the stomach. Interestingly, some peptides not only have antibacterial effects but also have immunomodulatory effects [27,28]. Does the SAMP-12aa screened in this study have both antibacterial and immunomodulatory effects by targeting the Foxp3 active center pocket? We used molecular docking to predict the binding of peptides to the active center pocket of Foxp3, and flow cytometry was used to determine the effect of the peptides on the expression level of the CD25 protein in CD4^+^ T cells. ELISA was used to determine the effect of SAMP-12aa on the expression level of the cytokine TGF-β, which inhibits CD8^+^ T-cell activity, and IFN-γ, which activates CD8^+^ T-cell activity. Molecular docking technology found that the binding of SAMP-12aa to the active pocket of Foxp3 has high specificity and affinity. We found that SAMP-12aa could downregulate the expression of the CD25 protein. SAMP-12aa could downregulate the number of CD4^+^CD25^High^ Treg cells while also increasing the secretion level of IFN-γ, which enhances cellular immune activation, and reducing the secretion of immunosuppressive TGF-β molecules. SAMP-12aa has immunomodulatory properties that can alleviate immune tolerance induced by *H. pylori*. The research results demonstrate that SAMP-12aa has an immunomodulatory effect.

## 2. Results

### 2.1. Identification of the Strongest Antimicrobial Peptide (SAMP-12aa)

We examined the anti-*H. pylori* activities of 28 peptides. As seen from Table 1, there were large differences among these peptides. The strongest anti-*H. pylori* activity for all antimicrobial peptides was that of KRIVQRIKDVIR, and its minimum inhibitory concentration (MIC) was 8 µg/mL. KRIVQRIKDVIR was named SAMP-12aa. We further researched the antibacterial activity of SAMP-12aa with physiological saline and artificial gastric juice to prepare liquid culture medium. We found that the MIC of SAMP-12aa was also 8 µg/mL, which is the same as the MIC in Table 1. The minimum bactericidal concentration (MBC) of SAMP-12aa was 32 μg/mL in liquid medium prepared with physiological saline or artificial gastric juice. The results indicated that artificial gastric juice did not affect the antibacterial and bactericidal activity of SAMP-12aa.

### 2.2. Effect of Artificial Gastric Juice on H. pylori Growth and Antibacterial Activity of SAMP-12aa

To verify whether artificial gastric juice affects the survival of *H. pylori*, we examined the numbers of living *H. pylori* cells in PBS as a control and in artificial gastric juice (Table 2). The survival rate of *H. pylori* decreased over time. The reason was that *H. pylori* is a microaerophilic bacterium, and a high concentration of oxygen has a certain toxic effect on it. We found that artificial gastric juice had little effect on the survival of *H. pylori*. In addition, its effect on the activity of *H. pylori* was only slight. This finding demonstrated that *H. pylori* was highly adapted to artificial gastric juice.

### 2.3. Effect of Artificial Gastric Juice on Bactericidal Kinetics

**Figure 1:**
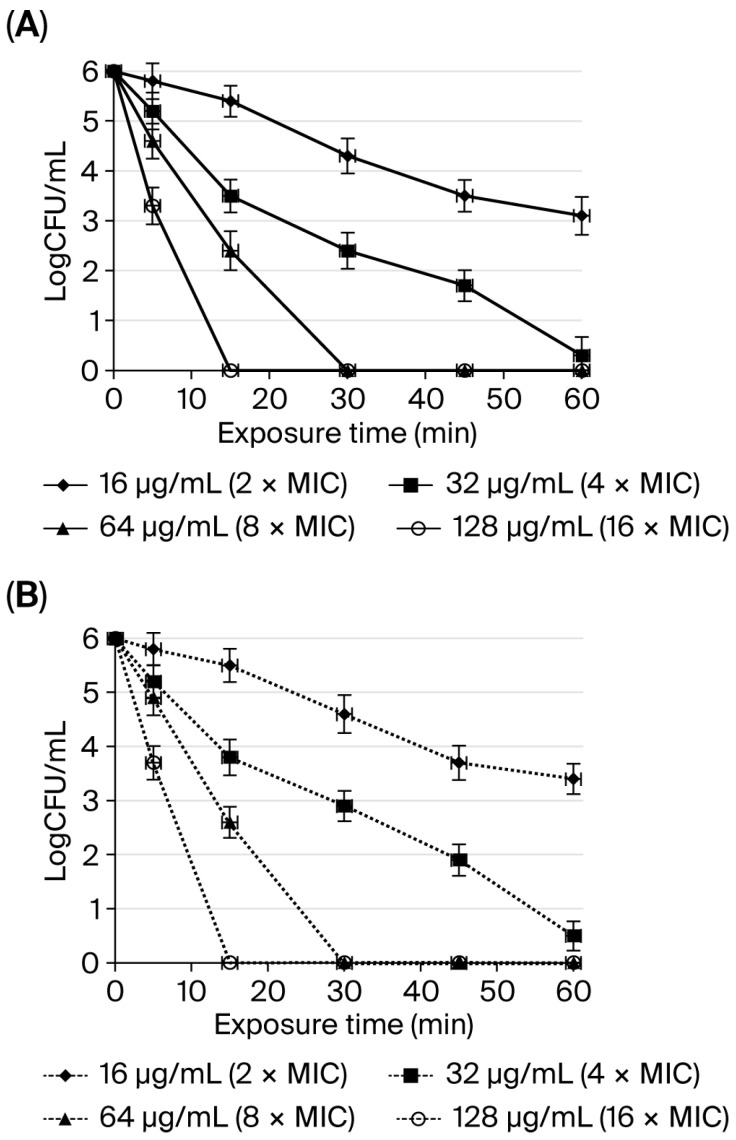
Comparison of the anti-*H. pylori* activity of SAMP-12aa prepared with physiological saline (**A**) or artificial gastric juice (**B**). The numbers of living bacteria (CFU) were measured in 10^6^ CFU/mL *H. pylori* solutions treated with different concentrations of SAMP-12aa and incubated in a warm bath at 37 °C for 0, 5, 15, 30, 45, and 60 min of exposure time, diluted by serial 10-fold dilutions, and plated on solid agar medium for CFU counts.

The bactericidal kinetics of SAMP-12aa against *H. pylori* ATCC43504 were compared between peptides prepared with physiological saline or artificial gastric juice. At 16 μg/mL (2 MIC) and 5 min, 15 min, 30 min, 45 min and 60 min exposure times, the numbers of living bacteria (CFU) in physiological saline or artificial gastric juice, respectively, differed (*p* > 0.05). At 32 μg/mL (4 MIC = MBC) and 5 min, 15 min, 30 min, 45 min, and 60 min exposure times, the numbers of living bacteria (CFU) in physiological saline or artificial gastric juice differed (*p* > 0.05). At 64 μg/mL (8 MIC = 2 MBC) and 5 min and 15 min exposure times, the numbers of living bacteria (CFU) in physiological saline and artificial gastric juice, respectively, differed (*p* > 0.05). At 128 μg/mL (16 MIC = 4 MBC) and 5 min of exposure time, the numbers of living bacteria (CFU) in physiological saline and artificial gastric juice, respectively, differed (*p* > 0.05). As shown in Figure 1 and according to the above data, the results indicated that the effect of artificial gastric juice on the bactericidal kinetics of SAMP-12aa was not obvious. SAMP-12aa showed good anti-*H. pylori* activity in gastric juice.

### 2.4. Effect of SAMP-12aa on Outer Membrane Permeability

**Figure 2:**
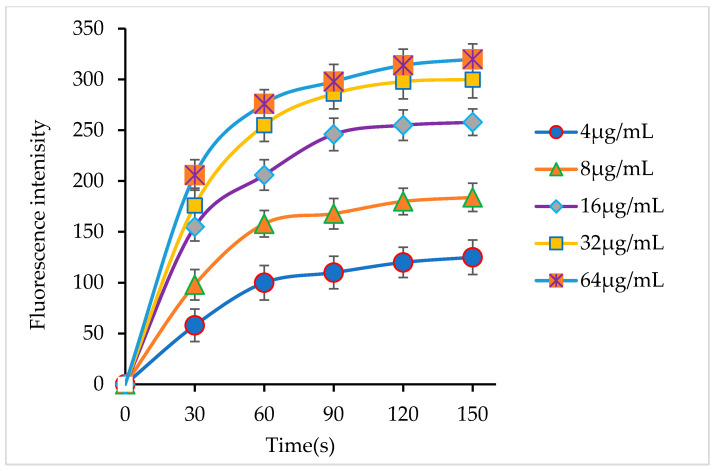
Effects of SAMP-12aa on permeability of the *H. pylori* outer membrane. SAMP-12aa at different concentrations can increase membrane permeability as shown by the change in the fluorescence intensity with time.

*H. pylori* is a Gram-negative bacterium with an outer membrane structure. The outer membrane of bacteria prevents a drug from entering the cell and causing damage. If antibacterial agents have the ability to penetrate the outer membrane, they will have a better bactericidal effect. NPN shows weaker fluorescence in an aqueous solution than in a hydrophobic environment. If SAMP-12aa has the ability to enhance the permeability of the outer membrane of *H. pylori*, the fluorescence intensity of the NPN probe will increase. In this study, we found that the intensity of the fluorescence of NPN was obviously enhanced when the *H. pylori* suspension contained SAMP-12aa, and the increase in fluorescence showed dose dependence, as shown in Figure 2. The findings demonstrated that SAMP-12aa possesses the ability to permeabilize the outer membrane of *H. pylori*.

### 2.5. Effect of SAMP-12aa on Cell Membrane Integrity

**Figure 3:**
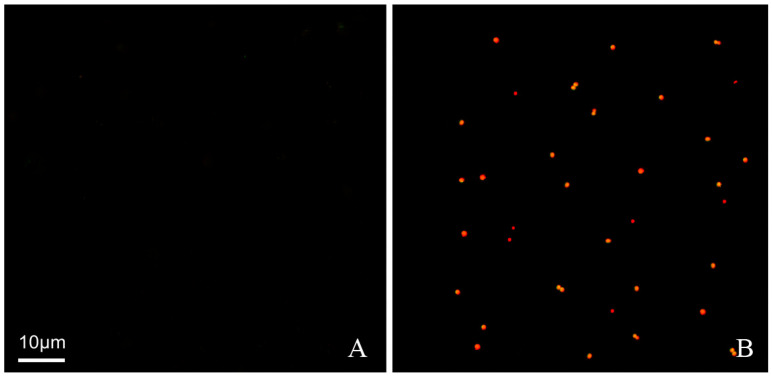
Effect of SAMP-12aa on *H. pylori* membrane permeabilization. *H. pylori* were incubated with (**A**) PBS (a control) or (**B**) 50 M PI and 0.3 mg/mL SAMP-12aa for 90 min at room temperature under microaerophilic conditions. The cells were observed using fluorescence confocal microscopy. (**A**) No fluorescence was observed, indicating that PI could not penetrate into cells. (**B**) A few red fluorescent dots could be observed, demonstrating that PI could enter cells and bind to DNA.

Destruction of the integrity of the bacterial inner membrane, i.e., the cell membrane, will lead to bacterial death. At the same time, the selective penetration of bacteria will also be inhibited. Propidium iodide (PI) can only penetrate dead cells and cannot penetrate living cells. PI penetrates into cells and then binds to DNA and emits red fluorescence. In this study, a visual method could be used to prove whether SAMP-12aa destroyed the integrity of the *H. pylori* cell inner membrane and led to bacterial death. When cell death occurs, the penetration of PI is promoted. As shown in Figure 3, no red fluorescent spots were observed (Figure 3A) when *H. pylori* was incubated with PBS as a control. However, when *H. pylori* were incubated with SAMP-12aa, there were obvious red fluorescent spots as shown in Figure 3B. The results demonstrated that SAMP-12aa has the ability to destroy the integrity of the bacterial cell membrane, so it has bactericidal efficacy. This bactericidal mechanism for SAMP-12aa does not easily induce drug resistance.

### 2.6. Resistance Assay of SAMP-12aa

**Figure 4:**
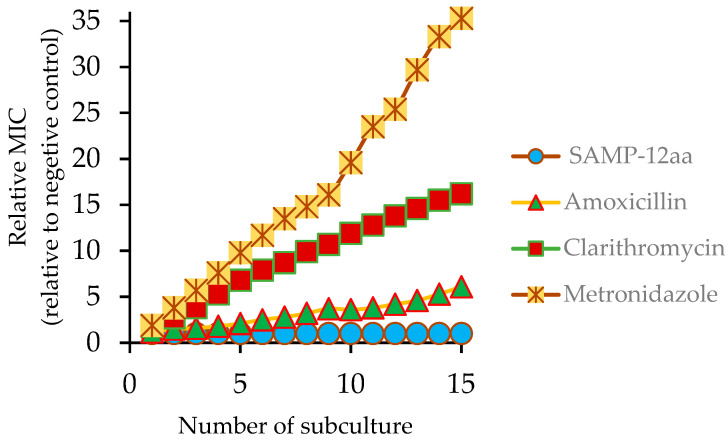
In vitro development-of-resistance studies. Evolution of MICs after successive exposures of *H. pylori* to subinhibitory concentrations of an antimicrobial agent. After 15 serial passages, the relative MIC was the normalized ratio of the MIC obtained for a given subculture to the MIC obtained for first-time exposure.

To evaluate whether the antimicrobial agents could induce resistance, *H. pylori* was repeatedly exposed to subinhibitory concentrations of antimicrobial agents. As shown in Figure 4, SAMP-12aa did not induce resistance of *H. pylori*. The relative MIC of SAMP-12aa against *H. pylori* remained stable for 15 consecutive subcultures. However, the first-line antibiotics for the treatment of *H. pylori* infection induced *H. pylori* resistance to them to different degrees. The resistance of *H. pylori* to metronidazole was particularly obvious after repeated administration. The MIC of metronidazole for *H. pylori* increased 35-fold, followed by clarithromycin, with its MIC for *H. pylori* increasing by 16-fold. *H. pylori* also induced resistance to amoxicillin, with its MIC for *H. pylori* increasing by 6-fold. Interestingly, *H. pylori* strains that were resistant to antibiotics were still sensitive to SAMP-12aa. The results showed that SAMP-12aa could be used to treat antibiotic-resistant *H. pylori* infection.

### 2.7. The Therapeutic Index of SAMP-12aa

In this study, the geometric mean (GM) was obtained from the MIC values against 6 different *H. pylori* bacterial strains. The minimal hemolytic concentration (MHC) was determined as the concentration at which the peptide can induce 10% hemolysis of human red blood. The therapeutic index (PI) was calculated as the ratio of the MHC (μg/mL) to the GM (μg/mL). The values of GM, MHC, and TI for SAMP-12aa are shown in Table 3. The results indicated that SAMP-12aa has greater antimicrobial specificity. The hemolysis concentration of SAMP-12aa reached 216 μg/mL, which was a low hemolytic activity (high MHC). The GM was 8.5 μg/mL, indicating high antimicrobial activity and low GM. Therefore, the antimicrobial peptide SAMP-12aa is an ideal antibacterial agent for the treatment of *H. pylori* infection.

### 2.8. Cytotoxicity of SAMP-12aa

**Figure 5:**
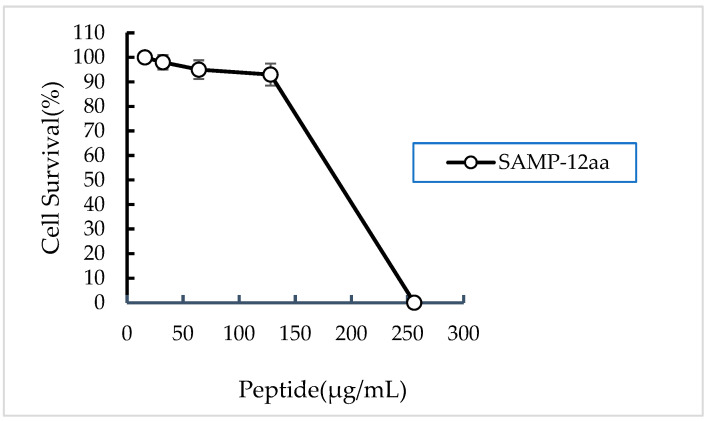
The cytotoxicity of SAMP-12aa against human gastric adenocarcinoma cells.

The cytotoxicity of SAMP-12aa against human gastric adenocarcinoma cells (ATCC; CRL-1739) was evaluated using a standard MTT assay. The toxicity of SAMP-12aa on living cells was assessed by measuring insoluble blue-purple formazan crystals. This substrate could only be produced in living cells, in which succinate dehydrogenase in mitochondria reduces exogenous MTT, but dead cells have no such function. As shown in Figure 5, the results demonstrated that SAMP-12aa was nontoxic toward CRL-1739 cells at 128 μg/mL, and there were more than 90% viable cells at this concentration. The results suggested that SAMP-12aa is a good candidate for development and application in the treatment of *H. pylori* infection owing to its low toxicity to eukaryotic cells.

### 2.9. Results of the Interaction Between SAMP-12aa and Foxp3 According to Molecular Docking Analysis

**Figure 6:**
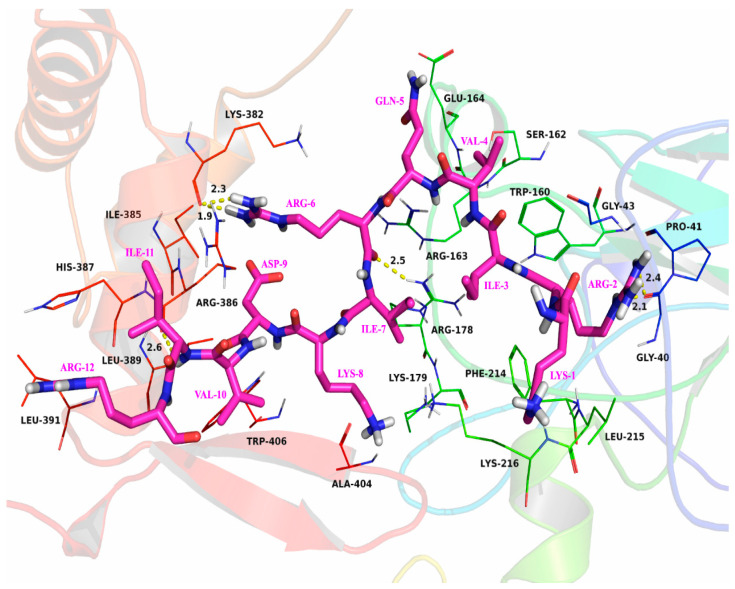
The molecular docking analysis of SAMP-12aa (amino acid, in purple red). Foxp3 protein molecule active pocket (amino acid, in black) interaction.

The characteristics and affinity of the interaction between SAMP-12aa and Foxp3 protein active pockets were predicted using molecular docking analysis. As shown in Figure 6, SAMP-12aa is tightly bound to the surface of the Foxp3 active pocket. The Ile-3 and Val-4 amino residues of SAMP-12aa are located in the hydrophobic pocket composed of Trp-160 and Phe-214 amino acid residues of Foxp3 and form stable hydrophobic interactions. Moreover, Val-10 and Ile-11 of SAMP-12aa are located in another hydrophobic pocket composed of Leu-389, Leu-391, and Trp-406 of Foxp3 and form stable hydrophobic interactions. Further detailed analysis revealed that Arg-2 of SAMP-12aa could interact with Gly-40 of Foxp3, forming two hydrogen bonds measuring 2.1 Å and 2.4 Å. Similarly, Arg-6 of SAMP-12aa can form hydrogen bonds measuring 2.5 Å, 1.9 Å, and 2.3 Å with the Arg-178 and Lys-382 amino acid residues of Foxp3. In addition, the Ile-11 amino acid residue of SAMP-12aa can form a hydrogen bond measuring 2.6 Å with Arg-386 of Foxp3. In this binding mode, SAMP-12aa forms a stable complex with Foxp3. The results suggested that SAMP-12aa could affect the transcriptional activity of the transcription factor Foxp3, then further affecting the expression level of the CD25 protein molecule.

### 2.10. Effect of SAMP-12aa on the Development and Differentiation of Treg Cells Induced by H. pylori

**Figure 7:**
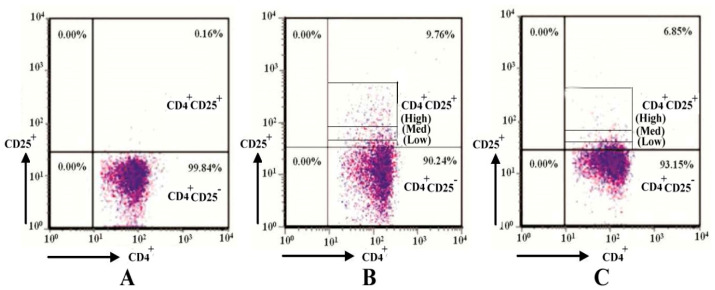
The effect of SAMP-12aa on immune tolerance induced by *H. pylori*. (**A**) CD4^+^Foxp3^-^CD25^-^T cells analyzed using magnetic cell sorting. (**B**) *H. pylori* promotes the development and differentiation of CD4^+^ Foxp3^+^CD25^Med-Low^ and CD4^+^ Foxp3^+^CD25^High^ Treg cells. (**C**) SAMP-12aa hinders the development and differentiation of CD4^+^Foxp3^+^ CD25^Med-Low^ and CD4^+^ Foxp3^+^CD25^High^ Treg cells induced by *H. pylori*.

High-purity (>98%) CD4^+^Foxp3^-^ CD25^-^ T cells can be isolated from peripheral blood cells using magnetic cell sorting technology and assessed with fluorescence-activated cell sorting technology (FACS) (as shown in Figure 7A). *H. pylori* virulence factors can enhance the development and differentiation of CD4^+^Foxp3^+^CD25^+^ Treg cells, especially CD4^+^Foxp3^+^ CD25^High^ Treg cells with an immunosuppressive effect, through the presentation of antigens by dendritic cells. The population of Treg cells obviously increased from 0.16% to 9.76% (as shown in Figure 7B). The effects of SAMP-12aa on the development and differentiation of CD4^+^Foxp3^+^CD25^High^ Treg cells with immunosuppressive activity were explored. The number of CD4^+^Foxp3^+^CD25^High^ Treg cells significantly decreased. The overall number of Treg cells also obviously decreased from 9.76% to 6.85% (as shown in Figure 7C). This study demonstrates that SAMP-12aa exerts an immune regulatory function by acting on the transcription factor Foxp3.

### 2.11. ELISA Results

CD4^+^Foxp3^-^CD25^-^ T cells differentiate into CD4^+^Foxp3^+^CD25^High^ Treg cells induced by *H. pylori* antigens. High expression of the CD25 protein promotes the expression of CTLA-4 on the surface of T cells and the secretion of the cytokine TGF-β, which inhibits CD8^+^ T-cell activation. SAMP-12aa targets the Foxp3 active center, leading to a decrease in the population of CD4^+^Foxp3^+^CD25^High^ Treg cells. The expression level of CTLA-4 decreases, the secretion of TGF-β decreases (*p* < 0.01), and the secretion level of IFN-γ increases (*p* < 0.01) (as seen from Table 4). These changes are beneficial for promoting the activation of CD8^+^ T cells into effector T cells.

## 3. Discussion

The aim of this study was to find a new peptide with anti-*H. pylori* activity in gastric juice. Short peptides can save the cost of treatment for drug development. We designed 28 short peptides with 12 amino acids based on the N-terminus of 9 amino acids originating from LL-37 and designed 3 amino acids at its C-terminus (Table 1). The peptide with the strongest anti-*H. pylori* activity was screened out by testing the MIC of anti-*H. pylori* activity. Through further analysis, we found that these peptides were all cationic peptides. When the charge of the cationic polypeptide was less than +5, the antibacterial activity increased with increasing positive charge number. This result was consistent with that of a previous report [29]. The positively charged amino acids were mainly Arg and Lys, but the position of Arg and Lys in the peptide also affected the antibacterial activity. This result was also consistent with that of a previous report [30]. Table 1 shows that, if there are Arg and Lys amino acids in the addition of three amino acid residues, the antibacterial activity is enhanced. At the same time, we found that hydrophobic groups play a key role in the process of antibacterial peptides inserting into the cell membrane. Increasing the hydrophobicity of antibacterial peptides can enhance their antibacterial activity. The effect of hydrophobicity on their activity can be evaluated through changing the numbers of Leu, Ile, and Val in the peptide chain [31]. A greater positive charge was not correlated with antibacterial activity. The polypeptide had a more positive charge after adding three Arg residues (RRR), with relative few hydrophobic amino acids, and its antibacterial activity was not the strongest. These results may provide a theoretical basis for the design of antibacterial peptides in the future. We have further confirmed that antimicrobial peptides with the same structural characteristics also have antibacterial activity in our other studies.

*H. pylori* are becoming increasingly resistant to existing antibiotics [3,32,33]. Because of the resistance of *H. pylori* to antibiotics, the treatment plan for *H. pylori* has changed greatly [34,35]. With the increasing number of antibiotic-resistant strains, it is necessary and urgent to find new antibacterial drugs to treat *H. pylori* infection. Antimicrobial peptides may be suitable candidates for treating this infection [33]. Antimicrobial peptides are also expressed in the surface epithelium of the gastrointestinal tract. The expression of LL-37 increased in the stomach of patients with *H. pylori* infection [7]. Thus, antimicrobial peptides play a certain role in the host defense against *H. pylori*. Unfortunately, most patients with *H. pylori* infection are in a chronic state of infection, and LL-37 did not remove *H. pylori* from the infected stomach. A strongly acidic environment and pepsin can destroy the original structure of LL-37, which becomes a small peptide in simulated gastric juice [8]. Furthermore, the antibacterial activity of LL-37 was affected by these conditions. Therefore, it is necessary to design and modify the existing antimicrobial peptides to ensure the antibacterial activity of antimicrobial peptides in the gastric environment, and antimicrobial peptides must be protected from gastric acid and pepsin degradation. In this study, one of the most potent anti-*H. pylori* peptides (SAMP-12aa) was obtained from 28 peptides that were stable in the gastric environment. We found that SAMP-12aa, with the strongest anti-*H. pylori* activity, still maintained the original anti-*H. pylori* activity in the simulated artificial gastric juice (Table 3). Because of the emptying of the stomach, the retention time of drugs in the stomach is short. If a drug can kill *H. pylori* in a short time, it will have great value in clinical applications. In this study, we found that SAMP-12aa had good bactericidal kinetics, killing *H. pylori* at 128 μg/mL and 64 μg/mL after 15 min and 30 min, respectively. More importantly, artificial gastric juice had little effect on the bactericidal kinetics of SAMP-12aa (Figure 1).

The antibacterial mechanism of traditional antibiotics against *H. pylori* is to inhibit the activity of cell wall synthase or the synthesis of functional proteins. In this way, it is easy for bacteria to inactivate antibiotics through gene mutation and produce drug resistance. Positively charged polypeptides combine with negatively charged bacterial cell walls to increase the permeability of bacterial cell membranes to kill bacteria. Bacteria are not susceptible to drug resistance. In this study, we found that SAMP-12aa has a strong ability to penetrate and damage the bacterial cell membrane (Figure 2 and Figure 3). At the same time, we found that SAMP-12aa did not induce the production of drug-resistant strains by inducing drug resistance in vitro (Figure 4). Cholesterol is present in eukaryotic cell membranes but not in prokaryotic cell membranes. This structural property leads to the selective toxicity of some antimicrobial peptides to prokaryotic cell membranes, such as bacteria, and almost no toxicity to eukaryotic cell membranes, such as human cells [36]. However, other antimicrobial peptides display toxicity to both prokaryotic and eukaryotic cells, and LL-37 is toxic to human erythrocytes [37]. If the antimicrobial peptide has prokaryotic selectivity, it is relatively safe at high drug concentrations and is beneficial for killing bacteria. The therapeutic index has been widely used to evaluate the prokaryotic selectivity of antimicrobial peptides [38,39,40,41]. These parameters are identified by the ratio of MHC (the minimal hemolytic activity) and GM (the geometric mean MIC of six microbial strains). A relatively large therapeutic index indicates greater antimicrobial specificity. In this study, we found that SAMP-12aa had a relatively large therapeutic index value (as seen from Table 4). To further confirm the cytotoxicity of antimicrobial peptides to human cells, we used the standard MTT analysis method to detect the cytotoxicity of SAMP-12aa on human gastric adenocarcinoma CRL-1739 cells. The results showed that SAMP-12aa had no toxicity to CRL-1739 cells at 128 μg/mL (Figure 5). Therefore, SAMP-12aa has the potential to be developed as an anti-*H. pylori* drug.

CD4^+^ T cells with medium or low levels of CD25 (CD25^med-low^) protein expression exhibit effector T-cell and memory T-cell functions without immune-suppressive regulatory function, and CD4^+^CD25^High^ Treg cells have immunosuppressive functions [42]. Compared with noninfected individuals, *H. pylori*-infected individuals exhibit significantly increased populations of CD4^+^CD25^High^ Treg cells in gastric mucosal tissues, which suppress immune responses and lead to the sustained colonization of *H. pylori* in the stomach [20]. Foxp3 is an important transcription factor for the expression of the CD25 protein, and studies have shown that the protein level of Foxp3 directly affects the expression level of the CD25 protein [25,26]. The inhibition of Foxp3 transcription factor activity may lead to the downregulation of CD25 protein expression levels, thereby reducing the development and differentiation of CD4^+^ T cells toward CD4^+^CD25^High^ Treg cells. In medicine, some larger molecular substances, such as peptides or proteins (e.g., insulin, growth hormone, calcitonin, posterior pituitary hormones, and interleukin-2), have been used as therapeutic drugs. Peptide drugs mainly exert their effects through high affinity and targeting. Foxp3 binds to other transcription factors and regulates the expression of the CD25 protein [25,26], thereby promoting the differentiation of CD4^+^ T cells into regulatory T cells. Specific targeting of the active center of Foxp3 by SAMP-12aa affects the binding of Foxp3 to other transcription factors, thereby downregulating the expression level of CD25 protein. The population of CD4^+^CD25^High^ Treg cells significantly decreased. Peptides bind to the active center target of Foxp3 with specific and high affinity. The targeting of the active center of Foxp3 by SAMP-12aa can be achieved through high-throughput screening techniques, which are widely recognized as the gold standard for discovering bioactive substances in the field of new drug discovery [41,42,43,44,45]. However, this screening technology is expensive to establish and maintain, which hinders its widespread application. Computer-assisted molecular docking technology has been widely applied in the prediction and discovery of new active substances [44]. Molecular docking technology was used to predict the binding of SAMP-12aa to the active pocket of Foxp3 in this study. Its binding has high specificity and affinity, as shown in Figure 7. Flow cytometry revealed that the population of CD4^+^CD25^High^ Treg cells induced by *H. pylori* antigens was decreased when SAMP-12aa was combined with Foxp3, as shown in Figure 7. The high expression of CTLA-4 protein molecules on the surface of CD4^+^CD25^High^ Treg cells competes with CD8^+^ T-cell surface CD28 to bind to CD80/CD86 protein molecules on antigen-presenting cells, inhibiting the second signal of effector T-cell activation and leading to the ineffective production of cytotoxic T cells. *H. pylori* has been shown to have intracellular bacterial characteristics in gastric mucosal epithelial cells [46], and the production of antibodies by humoral immunity cannot effectively eliminate its colonization in the gastric mucosa; only cellular immunity can eliminate its colonization in the gastric mucosa [47]. This study revealed that SAMP-12aa downregulates the expression of the CD25 protein, which is beneficial for promoting cellular immune function. We further examined cytokines and reported that *H. pylori* induced the production of CD4^+^CD25^High^ Treg cells with immunosuppressive activity, which also highly expressed the immunosuppressive cytokine TGF-β. SAMP-12aa downregulates the number of CD4^+^CD25^High^ Treg cells while also increasing the secretion level of IFN-γ (*p* < 0.01), which enhances cellular immune activation, and reducing the secretion of immunosuppressive TGF-β molecules (*p* < 0.01), as seen from Table 4. In summary, SAMP-12aa has immunomodulatory properties that can alleviate immune tolerance induced by *H. pylori*, which is beneficial for the immune system to clear *H. pylori* and colonization in the stomach.

## 4. Materials and Methods

### 4.1. Design of Short Peptides and Chemical Synthesis

The 18–26 amino acid residues KRIVQRIKD-NH2 of LL-37 are stable in the presence of acidity and pepsin, but this peptide’s antimicrobial activity was not determined experimentally. It was found that only the peptide with 12 amino acid residues exhibited better antimicrobial ability [10]. Therefore, in this study, 28 peptides with 12 amino acid residues were designed using a C-terminal method of nine amino acid residues supplemented with three amino acid residues. We designed 28 peptides (Table 1) that could not be degraded under low-pH conditions and in the presence of pepsin through an online network tool, https://web.expasy.org/peptide_cutter/ (accessed on 10 September 2024) for conducting analysis. To ensure that the peptide has better membrane penetration properties, the net positive charge carried by it cannot be less than +5. As shown in Table 1, all peptides were synthesized by Sangon Biotech Co., Ltd. (Shanghai, China) with a purity of 95%, as detected by high-performance liquid chromatography.

### 4.2. H. pylori Strains and Culture

*H. pylori* ATCC 43504 (NCTC 11637), *H. pylori* ATCC 700392 (26659), *H. pylori* ATCC 63629 (NCTC 11639), *H. pylori* SS1, *H. pylori* gastric ulcer clinical strain, and *H. pylori* gastric cancer clinical strain were used and preserved in our laboratory in this study. *H. pylori* were resurrected in a liquid medium of brain heart infusion (BHI) broth supplemented with 10% fetal calf serum (FCS). Then, *H. pylori* were plated and cultured in Colombian blood agar supplemented with 7% defibrinated sheep blood under microaerobic conditions at 37 °C. *H. pylori* were collected to conduct further experiments after 36 h to ensure that *H. pylori* were in the logarithmic growth phase.

### 4.3. Screening the Strongest Antimicrobial Peptide (SAMP-12aa)

To identify the strongest antimicrobial peptide, named SAMP-12aa, against *H. pylori* ATCC43504 activity from the 28 designed peptides, the minimum inhibitory concentration (MIC) was determined. The MICs of the designed peptides against *H. pylori* were measured by the double serial dilution method previously described by our group [5]. Log-phase bacteria were obtained by culturing *H. pylori* at 37 °C under microaerobic conditions for 36 h. A bacterial solution (1 × 10^8^ CFU/mL) was prepared. Peptides were diluted using the double dilution method from 256 µg/mL to 1 µg/mL (9 continuous dilutions in 9 test tubes and a tenth test tube without polypeptide as a control) with liquid medium. Ten microliters of bacterial solution were added to each test tube so that the concentration of test tube bacteria was approximately 10^6^ CFU/mL. Then, the cell cultures were shaken at 200 r/min for 36 h at 37 °C under microaerobic conditions. The minimum inhibitory concentration (MIC) was determined by confirming that the test tube containing the lowest drug concentration was optically clear, showing no visible turbidity or bacterial growth. The peptide with the strongest antimicrobial activity (SAMP-12aa) was determined for further study.

### 4.4. Survival Assay of H. pylori in Artificial Gastric Juice

For survival assays of *H. pylori* ATCC43504 in artificial gastric juice, log-phase *H. pylori* cultured in liquid medium at 37 °C in a microaerobic atmosphere for 24 h was centrifuged for 10 min at 3000 r/min to collect the bacterial cell pellet. The pellet was divided into two equal parts for a survival experiment and for use as a control. The cells were suspended in physiological saline or artificial gastric juice, and their concentrations were adjusted to 10^8^ CFU/mL for further application. The artificial gastric juice was prepared according to the reports of Leszczyńska et al. [8], and some modifications were made. The artificial gastric juice contained 2.05 g of NaCl, 8.3 g of proteose peptone, 0.6 g of KH_2_PO_4_, 0.37 g of KCl, 3.5 g of D-glucose, 0.11 g of CaCl_2_, 0.05 g of bile, 13.3 mg of pepsin, and 0.1 g of lysozyme per liter of gastric juice. Moreover, 5.5 g of urea was supplemented in artificial gastric juice to evaluate the role of *H. pylori* urease. The numbers of living *H. pylori* cells were determined at different time points of exposure to PBS or artificial gastric juice after 0 min, 30 min, and 60 min. Surviving bacterial cells were enumerated by plating appropriate dilutions of *H. pylori* cells onto Columbia blood agar plates and growth into a single colony. Then, colony counts were conducted on solid medium.

### 4.5. Anti-H. pylori Activity of SAMP-12aa in Artificial Gastric Juice

To prove whether the artificial gastric juice inhibited the antibacterial activity of SAMP-12aa, the MIC, minimum bactericidal concentration (MBC), and bactericidal kinetics of SAMP-12aa were determined in liquid and solid media prepared with physiological saline or artificial gastric juice. The bacterial solution and artificial gastric juice were prepared as described above. The MIC of SAMP-12aa was determined as described above. The minimum drug concentration of the test tube, in which no turbidity or bacterial growth was observed, was the minimum inhibitory concentration (MIC). Five microliters of mixed solution were taken from each of the above clarified tubes and inoculated on solid agar medium separately. Then, they were cultured under microaerobic conditions at 37 °C for 72 h. The minimum peptide concentration with no colony growth was the minimum bactericidal concentration (MBC) of SAMP-12aa.

### 4.6. Bactericidal Kinetics of SAMP-12aa in Artificial Gastric Juice

To further determine whether there was an effect of artificial gastric juice on the bactericidal kinetics of SAMP-12aa, SAMP-12aa were prepared with physiological saline or artificial gastric juice at concentrations of 2 × MIC, 4 × MIC, 8 × MIC, and 16 × MIC and incubated with 10^6^ CFU/mL *H. pylori* at 37 °C. The numbers of living cells were determined after 0 min, 5 min, 15 min, 30 min, 45 min, and 60 min of exposure to solutions of SAMP-12aa prepared with physiological saline or artificial gastric juice. Surviving bacterial cells were enumerated by plating appropriate dilutions of cells and counting bacterial colonies on solid medium.

### 4.7. Permeability Studies of the Outer Membrane

When the antibacterial mechanism of polypeptides is enhancing the permeability and destroying the integrity of the cell membrane, bacteria do not easily produce resistance to the peptides. In this study, the effect of SAMP-12aa on *H. pylori* ATCC43504 outer membrane permeabilization was determined using a fluorescent probe of hydrophobic 1-N-phenylnaphthylamine (NPN) [48,49]. The cell pellet was collected by centrifuging 50 mL of logarithmic-phase *H. pylori* cells at 5000× *g* for 10 min. Then, the cell pellet was suspended in 5 mM sodium HEPES buffer (pH 7.2) after washing three times with buffer. The optical density value for the suspension cells was adjusted to 0.5 at OD600. SAMP-12aa solutions of different concentrations at 4, 8, 16, 32, or 64 μg/mL were prepared. Four milliliters of each tested sample contained 1 mL of SAMP-12aa solution or 1 mL of HEPES buffer without peptide as a control, 2 mL of *H. pylori* cell suspension, and 1 mL of 40 mM NPN. The fluorescent intensity was measured at 30 s intervals from the beginning until the fluorescence density no longer increased, with a fluorescence spectrophotometer (F4600, Hitachi, Tokyo, Japan), using a 350 nm excitation wavelength and 420 nm emission wavelength. The fluorescent intensity increased when NPN penetrated the hydrophobic environment of the *H. pylori* outer membrane. The enhancement of the outer membrane permeability of *H. pylori* was due to the action of SAMP-12aa on the outer membrane. The experiments were carried out in triplicate.

### 4.8. Integrity Studies of the Cell Membrane

To determine whether SAMP-12aa damages the integrity of the *H. pylori* ATCC 43504 cell membrane, 4 mL of the tested sample containing 1 mL of the above prepared *H. pylori* cell suspension, 1 mL of 16 μg/mL SAMP-12aa peptide or 1 mL of PBS buffer (pH = 7.2, as a control), and 2 mL of 100 µM propidium iodide (PI) was kept in the dark at room temperature for 90 min after mixing thoroughly. The red fluorescence was measured with a fluorescence confocal microscope (LSM 710 Meta, Zeiss, Jene, Germany) at a 535 nm excitation wavelength and a 615 nm emission wavelength. PI can enter dead cells, but it cannot enter living cells. After entering the cell, it combines with DNA and emits red fluorescence. In this study, if SAMP-12aa could destroy the integrity of the bacterial cell membrane, resulting in cell death, red fluorescence would be produced.

### 4.9. Resistance Studies of SAMP-12aa

In this study, the *H. pylori* ATCC43504 strain was used to explore whether the SAMP-12aa peptide could induce resistance in vitro. Metronidazole, amoxicillin, and clarithromycin, which are conventional antibiotics used clinically for the treatment of *H. pylori* infection, were used as positive controls. The MICs of SAMP-12aa and the antibiotics were determined according to the above method. Resistance assays were conducted as described in previous reports [50]. *H. pylori* in the exponential growth stage was incubated in culture medium with subinhibitory concentrations of antimicrobial agents under microaerobic conditions for 36 h and then centrifuged to harvest cells that displayed nearly 50% growth inhibition activity. The cells were further diluted and adjusted to a concentration of 10^5^ CFU/mL with fresh medium and cultured again until 15 similar serial incubations were achieved. Culture medium without antimicrobial agent was used as a negative control. The evolution of MICs after successive exposures of *H. pylori* to subinhibitory concentrations of the antimicrobial agents was evaluated. If the relative MIC for each passage increased, the results showed that the drug induced the production of resistant strains. The relative MIC for each passage was determined by calculating the ratio of the MIC to the measured values of a given subculture compared to that obtained from the first exposure. To determine whether antibiotic-resistant *H. pylori* strains were sensitive to SAMP-12aa, we further detected the MIC of SAMP-12aa against resistant strains as described above. The statistical data were processed from triplicate analyses.

### 4.10. Hemolytic Studies of SAMP-12aa

If an antibacterial peptide drug has prokaryotic selectivity, exhibiting optimal antibacterial activity and killing bacterial cells but not mammalian cells, this peptide drug will have good therapeutic potential and relative clinical safety. The therapeutic index (TI) reflects the prokaryotic selectivity of polypeptides [37,38,39,40,41]. The TI of a polypeptide was determined by calculating the ratio of the value of the minimal hemolytic concentration (MHC) to the geometric mean (GM) of MIC values obtained against six different *H. pylori* strains. The hemolytic activity of SAMP-12aa was determined according to a previously reported method [37,51]. Briefly, fresh human blood was collected to obtain human red blood cells (hRBCs) using the centrifugation method at 1000× *g* for 7 min. Then, the hRBCs were washed 3 times carefully and fully with PBS (43 mM Na_2_HPO_4_, 11 mM KH_2_PO_4_, and 80 mM NaCl). A 4% (*w*/*v*) hRBC suspension was prepared using the above hRBCs resuspended in PBS. Five different concentrations of SAMP-12aa were prepared with two-fold serial dilutions from 512 μg/mL to 32 μg/mL.

Then, 250 μL of different concentrations of SAMP-12aa and 250 μL of 4% *w*/*v* hRBC suspension were added to the same 0.5 mL centrifuge tube with sufficient mixing. In the same way, 250 μL of PBS (APBS) without SAMP-12aa and 250 μL of 4% *w*/*v* hRBC suspension were added to the same 0.5 mL centrifuge tube as the negative control. Then, 250 μL of 0.1% Triton X-100 (Atriton) and 250 μL of 4% *w*/*v* hRBC suspension were added to the same 0.5 mL centrifuge tube as the positive control. All mixtures were incubated with the water bath method at 37 °C for 1 h. Two hundred microliters of supernatant was collected from each centrifuge tube by centrifugation at 1000× *g* for 5 min and then added to the wells of a 96-well plate. The supernatant absorbance was measured at 414 nm with a microplate reader. The absorbance value of PBS (A_PBS_) was identified as zero hemolysis. The absorbance value of the supernatant of 0.1% (*v*/*v*) Triton X-100-lysed hRBCs (A_Triton_) was established as 100% hemolysis. The hemolytic activity of SAMP-12aa was calculated as the percentage of hemolysis according to the following formula: hemolysis (%) = (A_sample_ − A_PBS_)/(A_Triton_ − A_PBS_) × 100. Relatively large values indicated greater prokaryotic selectivity and antimicrobial specificity. For accurate determination of the MIC of peptides against six different strains of *H. pylori*, SAMP-12aa was diluted in increments of 1 μg/mL from 4 μg/mL to 12 μg/mL. The MIC was determined as described above.

### 4.11. Cytotoxicity Studies of SAMP-12aa

Human gastric adenocarcinoma cells (ATCC; CRL-1739) were used to determine the cytotoxicity of SAMP-12aa. A standard MTT proliferation assay was used to determine the cytotoxicity of SAMP-12aa against CRL-1739 cells. The CRL-1739 cells were cultured in RPMI-1640 culture medium supplemented with 10% fetal bovine serum in 5% CO_2_ at 37 °C for 3–5 days. The adherent cells were treated to form dispersed single cells by trypsin digestion, and then, the cell pellet was collected after centrifugation at 1000× *g* for 4 min. A 1 × 10^5^/mL cell suspension was prepared with culture medium containing 10% fetal bovine serum. Then, 200 μL of cell suspension was added to each well of a 96-well plate, and the plates were cultured to form attached cells at 37 °C in 5% CO_2_ for 24 h. Forty microliters of SAMP-12aa solution with serial 2-fold dilutions from 256 μg/mL to 16 μg/mL with RPMI-1640 culture medium was added to the wells. The wells only containing 40 μL of RPMI-1640 culture medium and no SAMP-12aa were used as a control. The plates were allowed to further culture for 48 h. Then, 20 μL of 5 mg/mL MTT was added to each well and mixed carefully and thoroughly. The plates were incubated at 37 °C for 4 h. The supernatant of each well was removed carefully. Then, 150 μL of dimethyl sulfoxide (DMSO) was added to each well to dissolve the insoluble formazan crystals produced by MTT. A microplate ELISA reader was used to measure the absorbance of fully dissolved formazan crystals at 550 nm. The following formula was used to calculate the CRL-1739 cell survival rate: survival (%) = (A_550_ of SAMP-12aa-treated cells/A_550_ of SAMP-12aa-untreated cells) × 100.

### 4.12. Prediction of the Interaction Between Foxp3 and SAMP-12aa Using Molecular Docking Analysis

We used the transcription factor Foxp3 (PDB ID: 3QRF) as a template, and the software MODELLER 9.18 http://salilab.org/modeller/ (accessed on 8 September 2024) was used for homology modeling. The three-dimensional structure of Foxp3 was obtained, after which PyMOL 1.7.6 was used to construct the three-dimensional structure of SAMP-12aa. Afterward, SYBYL-X 2.0 software was used to optimize the three-dimensional structure. The three-dimensional structure of SAMP-12aa was obtained. Foxp3 and the small peptide were transformed into PDBQT format using AutoDockTools 1.5.6 [52]. AutoDock Vina 1.1.2 [53,54] was used for molecular docking analysis.

### 4.13. Effects of SAMP-12aa on the Development of Regulatory T Cells Induced by H. pylori

Analysis revealed that *H. pylori* promotes immune tolerance through increasing the development and the differentiation of CD4^+^Foxp3^+^ CD25^High^ Treg cells by the transcription factor Foxp3. To obtain dendritic cells (DCs), peripheral blood samples were obtained from voluntary donors who were not infected with *H. pylori*, and the study was approved by the Ethics Committee of Anhui University of Science and Technology. Differential centrifugation was used to separate blood samples and obtain peripheral blood mononuclear cells (PBMCs) with lymphocyte separation medium (Serumwerk Bernburg AG, Bernburg, Germany). PBMCs were washed twice with PBS, after which CD14^+^ cells were obtained from PBMCs through magnetic bead positive selection. A total of 3 × 10^6^ CD14^+^ cells were seeded into each well of six-well plates and incubated with DC medium contain RPMI 1640 supplemented with 1.0% human AB serum, 1.0% penicillin–streptomycin, 1.0% sodium pyruvate, 50 ng/mL granulocyte–macrophage colony-stimulating factor (GM-CSF), and 10 ng/mL interleukin 4 (IL-4) in a humidified chamber at 37 °C and 5% CO_2_ (these conditions were adapted to all of the following incubations). The fresh culture was added to the medium containing the cytokines GM-CSF and IL-4 every 2 days. Six days later, peripheral blood samples were obtained again to prepare PBMCs, and CD4^+^CD25^-^ T cells were separated from PBMCs using magnetic beads. CD4^+^CD25^-^ T-cell fractions with content greater than 96% were determined using flow cytometry analysis. The supernatants of the above cultured DCs were discarded, and the cells were resuspended in fresh culture medium. After counting, the cells were divided into three different wells (6 wells each) and cultured in 24-well culture plates. In the first wells, CD4^+^ CD25^-^T cells were cocultured with DCs at a ratio of 2:1 (1 × 10^5^ T cells to 0.5 × 10^5^ DCs) and anti-CD3ε at a concentration of 1 μg/mL (Invitrogen). In the second wells, CD4^+^CD25^-^T cells were cocultured with DCs at a ratio of 2:1 (1 × 10^5^ T cells to 0.5 × 10^5^ DCs) and 10^4^ *H. pylori* antigens in RPMI 1640 supplemented with 10% fetal bovine serum and 10 ng/mL rIL-2 (R&D Systems), without anti-CD3ε. The antigen preparation method was as follows: *H. pylori* ATCC700392 (26659), which contains VacA (vacuolating cytotoxin) as an important antigen for inducing immune tolerance, was cultivated in blood agar medium under microaerophilic conditions at 37 °C for 3 days. After counting using optical density, 10^8^ *H. pylori* were killed with 0.2 mg of streptomycin and 200 U of penicillin per mL for 6 h. *H. pylori* antigens were prepared after ultrasonic fragmentation. In the third wells, according to the instructions of the Pierce Protein Transfection Reagent Kit (Thermo Scientific), 50 μg/mL SAMP-12aa was prepared with HEPES-buffered saline (HBS; 10 mM HEPES, 150 mM NaCl, pH 7.0), after which the peptide and protein/peptide transfection reagent complexes were prepared. A total of 1 × 10^5^ CD4^+^ CD25^-^ T cells were allowed to adhere to the wall and were subsequently washed twice with serum-free medium. An appropriate amount of peptide and transfection reagent complex (containing 2 μg of SAMP-12aa) was added to the 24-well plate, and the final volume of the Pierce Reagent/SAMP-12aa mixture was adjusted to 1000 μL with serum-free medium. The cells were incubated in a 5% CO_2_ incubator at 37 °C for 4 h, after which 1000 μL (one volume) of 20% serum-containing medium was directly added to a well containing 0.5 × 10^5^ DCs, 10^4^ *H. pylori* antigen, and 10 ng/mL rIL-2. After 5 days of coculture, the cells were stained first for CD4 (CD4^-^FITC clone RPA-T4; eBioscience), CD25 (CD25^-^PE clone BC96; eBioscience), and CD152 (CTLA-4-FITC clone 14D3; eBioscience) and, then, after fixation and permeabilization, for Foxp3 (Foxp3^-^APC clone PCH101; eBioscience). The percentage of CD4^+^Foxp3^+^CD25^+^ T cells was assessed by fluorescence-activated cell sorting (FACS) technology.

### 4.14. Detection of Cytokines

The supernatant from each coculture well was collected, and the expression levels of TGF-β and IFN-γ in the culture medium were detected using ELISA to determine the differentiation of different types of CD4^+^ T cells. ELISA was performed according to the instructions provided by the manufacturer (BD Biosciences) of the TGF-β and IFN-γ kits.

### 4.15. Statistical Analysis

Each experiment was conducted with independent, parallel triplicate samples. The average values of all the results were obtained from the above three independent experiments. Data are expressed as the mean ± standard deviation of the mean, with error bars representing the standard deviation. One-way analysis of variance (ANOVA) was used to determine the statistical significance of differences between measured samples and controls (no added SAMP-12aa antimicrobial agent). Differences between different samples with *p* < 0.05 were considered statistically significant.

## 5. Conclusions

In this study, SAMP-12aa, with 12 amino acids, was obtained based on 9 amino acid residues of LL-37. SAMP-12aa could maintain strong anti-*H. pylori* activity in artificial gastric juice. It was proven that SAMP-12aa was not degraded and inactivated by pepsin in gastric acid. SAMP-12aa had good bactericidal kinetics, killing *H. pylori* at 128 μg/mL and 64 μg/mL after 15 min and 30 min, respectively. Because of the emptying of the stomach, the retention time of drugs in the stomach is short, and this peptide could kill *H. pylori* in a sufficiently short time, having great value in clinical applications. At the same time, it was found that SAMP-12aa could enhance the permeability of the *H. pylori* extracellular membrane and destroy the integrity of the cell membrane; this bactericidal mechanism does not promote drug resistance in *H. pylori*. It was found that SAMP-12aa did not induce the production of drug-resistant strains according to drug resistance induction experiments in vitro. The results of hemolysis and cytotoxicity analysis showed that SAMP-12aa has a relatively high therapeutic index and hemolytic concentration. These data indicate that SAMP-12aa has good safety in clinical applications. In short, our results show that SAMP-12aa can be developed as a new drug for the treatment of *H. pylori* infection. Molecular docking prediction revealed that SAMP-12aa has high specificity and affinity for targeting Foxp3 active centers and binding to them. Flow cytometry analysis at the cellular level confirmed that SAMP-12aa has the ability to downregulate *H. pylori* antigen-induced CD4^+^CD25^-^T cells with high expression of CD25 protein and their differentiation into CD4^+^CD25^High^ Treg cells with immunosuppressive effects. Further confirmation was obtained through ELISA detection showing that SAMP-12aa downregulates the inhibitory cytokine TGF-β secreted by CD4^+^CD25^High^ Treg cells and upregulates the cytokine secretion level of IFN-γ, which can exert cellular immune activity. This study demonstrates that SAMP-12a has immunomodulatory functions, A new technological method is provided for the further use of SAMP-12aa in the prevention and treatment of *H. pylori* infection. Therefore, this study has certain theoretical significance and practical value.

## Figures and Tables

**Table 1:** Anti-*H. pylori* ATCC43504 strain activity of designed peptides.

Designed Peptides	MIC (μg/mL)	Designed Peptides	MIC (μg/mL)
1. KRIVQRIKDVIR	8	15. KRIVQRIKDIKP	32
2. KRIVQRIKDVGP	>256	16. KRIVQRIKDIIF	>256
3. KRIVQRIKDTAP	>256	17. KRIVQRIKDIEP	>256
4. KRIVQRIKDSVP	>256	18. KRIVQRIKDIAP	>256
5. KRIVQRIKDRVP	64	19. KRIVQRIKDIAE	>256
6. KRIVQRIKDRRR	32	20. KRIVQRIKDGPI	>256
7. KRIVQRIKDPSP	>256	21. KRIVQRIKDGKV	64
8. KRIVQRIKDPGP	>256	22. KRIVQRIKDGGP	>256
9. KRIVQRIKDMPP	>256	23. KRIVQRIKDGGM	>256
10. KRIVQRIKDMNP	>128	24. KRIVQRIKDGEG	>256
11. KRIVQRIKDIVP	>256	25. KRIVQRIKDERP	32
12. KRIVQRIKDIVQ	>256	26. KRIVQRIKDADA	>256
13. KRIVQRIKDIRI	64	27. KRIVQRIKDAAP	>256
14. KRIVQRIKDIPP	>256	28. KRIVQRIKDAEP	>256

**Table 2:** Survival of *H. pylori* ATCC43504 strain in artificial gastric juice.

Time (min)	Cell Survival			
	Control (PBS)		Artificial Gastric Juice	
	CFU/mL	%Survival	CFU/mL	%Survival
0	1.00 × 10^8^	100%	1.00 × 10^8^	100%
30	0.98 × 10^8^	98%	0.97 × 10^8^	97%
60	0.95 × 10^8^	95%	0.94 × 10^8^	94%

The number of surviving *H. pylori* cells was determined by cell colony counting.

**Table 3:** The therapeutic index (TI) of SAMP-12aa.

Peptide	MIC(μg/mL) of Six Different *H. pylori* Strains						GM (μg/mL)	MHC (μg/mL)	Therapeutic Index (MHC/GM)
	1	2	3	4	5	6			
SAMP-12aa	8	10	8	8	8	9	8.5	216	25.4

Strain 1: *H. pylori* ATCC 43504 (NCTC 11637); Strain 2: *H. pylori* ATCC 700392 (26659); Strain 3: *H. pylori* ATCC 63629 (NCTC 11639); Strain 4: *H. pylori* SS1; Strain 5: *H. pylori* gastric ulcer clinical strain; Strain 6: *H. pylori* gastric cancer clinical strain.

**Table 4:** The results of cytokine detection for TGF-β and IFN-γ.

Supernatant of Coculture Well	TGF-β (pg/mL)	IFN-γ (ng/mL)
CD4^+^T cells	18.7 ± 16.3	0.65 ± 0.53
CD4^+^T cells+*H. pylori*	71.3 ± 32.5 ^△^	0.36 ± 0.25 ^▲^
CD4^+^T cells+*H. pylori*+ SAMP-12aa	26.5 ± 18.6 ^△△^	1.83 ± 0.97 ^▲▲^

TGF-β: ^△^ *p* < 0.01 compared with CD4^+^T cells; ^△△^ *p* < 0.01 compared with CD4^+^T cells+*H. pylori*; INF-γ: ^▲^ *p* < 0.01 compared with CD4^+^T cells; ^▲▲^ *p* < 0.01 compared with CD4^+^T cells+*H. pylori.*

## Data Availability

The original contributions presented in this study are included in the article. Further inquiries can be directed to the corresponding authors.
